# Sleep EEG oscillations and synaptic plasticity in memory consolidation across NREM and REM sleep

**DOI:** 10.1080/19585969.2026.2744744

**Published:** 2026-10-15

**Authors:** Yuxuan Chen, Meimei Zhao, Xue Cao, Gao Zhou, Yu Zhang

**Affiliations:** a Department of Anesthesiology, Affiliated Hospital of Zunyi Medical University, Guizhou, Zunyi, China; b Key Laboratory of Anesthesia and Organ Protection of Guizhou Province, Zunyi Medical University, Guizhou, Zunyi, China

**Keywords:** Memory consolidation, sleep stages, neural oscillations, synaptic plasticity, REM sleep, NREM sleep

## Abstract

Based on a synthesis of recent advances, this review elucidates the distinct yet synergistic roles of non-rapid eye movement (NREM) and rapid eye movement (REM) sleep in memory consolidation. During NREM sleep, cortical slow oscillations (SOs) establish a temporal framework by alternating between UP and DOWN states, facilitating cross-regional communication. SOs phase-couple with thalamic spindles to coordinate hippocampal sharp-wave ripples (SWRs), thereby driving the reactivation and transfer of labile hippocampal memories to neocortical networks for long-term storage. Concurrent synaptic homeostasis optimises network efficiency through global downscaling while selectively strengthening memory-relevant connections. In REM sleep, theta oscillations synchronise limbic-prefrontal-hippocampal circuits, and ponto-geniculo-occipital (PGO) waves further integrate emotional and procedural memories. The cholinergically dominant, monoamine-depleted neurochemical milieu of REM sleep promotes dendritic spine remodelling and sparse neuronal reactivation, enabling affective decoupling and synaptic refinement. Despite progress, key challenges remain in understanding cross-frequency oscillatory coupling among slow oscillations, spindles, ripples and REM-specific rhythms, glial contributions and clinical translation. Future integration of high-density electrophysiology with computational approaches may yield mechanistic models and clinically relevant biomarkers for sleep-based cognitive monitoring and intervention.

## Introduction

Memory represents a core higher-order function of the brain, essential for adaptive responses to environmental demands. Through the dynamic interplay of encoding, consolidation and retrieval, experiential information is transformed into adaptive behavioural strategies, enabling organisms to continually optimise decision-making in ever-changing contexts (Diekelmann and Born 2010). The mechanisms underlying these processes form the foundation upon which innate predispositions and acquired experiences jointly shape the diversity of cognitive function (Blokland et al. 2008).

Memory encoding refers to the neurobiological process by which sensory inputs or internally generated thoughts are transformed into persistent neural representations-commonly termed memory traces. Consolidation denotes the time-dependent stabilisation of memories after acquisition, during which experience-related internal representations and their associated neurophysiological substrates undergo dynamic reorganisation. Retrieval is the process of reactivating or reconstructing memory traces associated with specific experiences, relying on the coordinated reconfiguration of the hippocampal-neocortical network.

Sleep is a naturally occurring, reversible physiological state characterised by diminished consciousness, elevated response thresholds and behavioural quiescence, regulated with remarkable precision by homeostatic mechanisms. By substantially reducing the processing of external information, sleep offers an optimal neurophysiological milieu for memory consolidation: newly encoded memories are integrated during sleep and stored for the long term (Rasch and Born 2013). Distinct stages of sleep contribute uniquely to the consolidation process, reflecting stage-specific biological mechanisms.

The present review aims to synthesise recent advances in how sleep-specific EEG oscillations shape memory consolidation across NREM and REM sleep. Rather than treating sleep stages as unitary and homogeneous, we adopt an event-based perspective that focuses on distinct oscillatory motifs—slow oscillations, spindles, hippocampal sharp-wave ripples, REM theta and PGO waves—and on their cross-frequency coupling as candidate mechanisms for systems-level memory reorganisation. By bringing together evidence from human EEG, intracranial recordings, animal models and computational work, we seek to clarify what has been gained beyond a mere accumulation of correlational findings: namely, a mechanistic framework that links characteristic sleep rhythms to specific operations such as hippocampo-cortical communication, synaptic renormalisation and emotional memory processing. We also highlight key unresolved issues and conceptual tensions, including the incomplete causal mapping between individual oscillatory events and particular memory functions, the interaction between neuronal, glial and neuromodulatory processes and the challenge of translating basic insights into clinically meaningful interventions (Figure 1).

**Figure 1.**
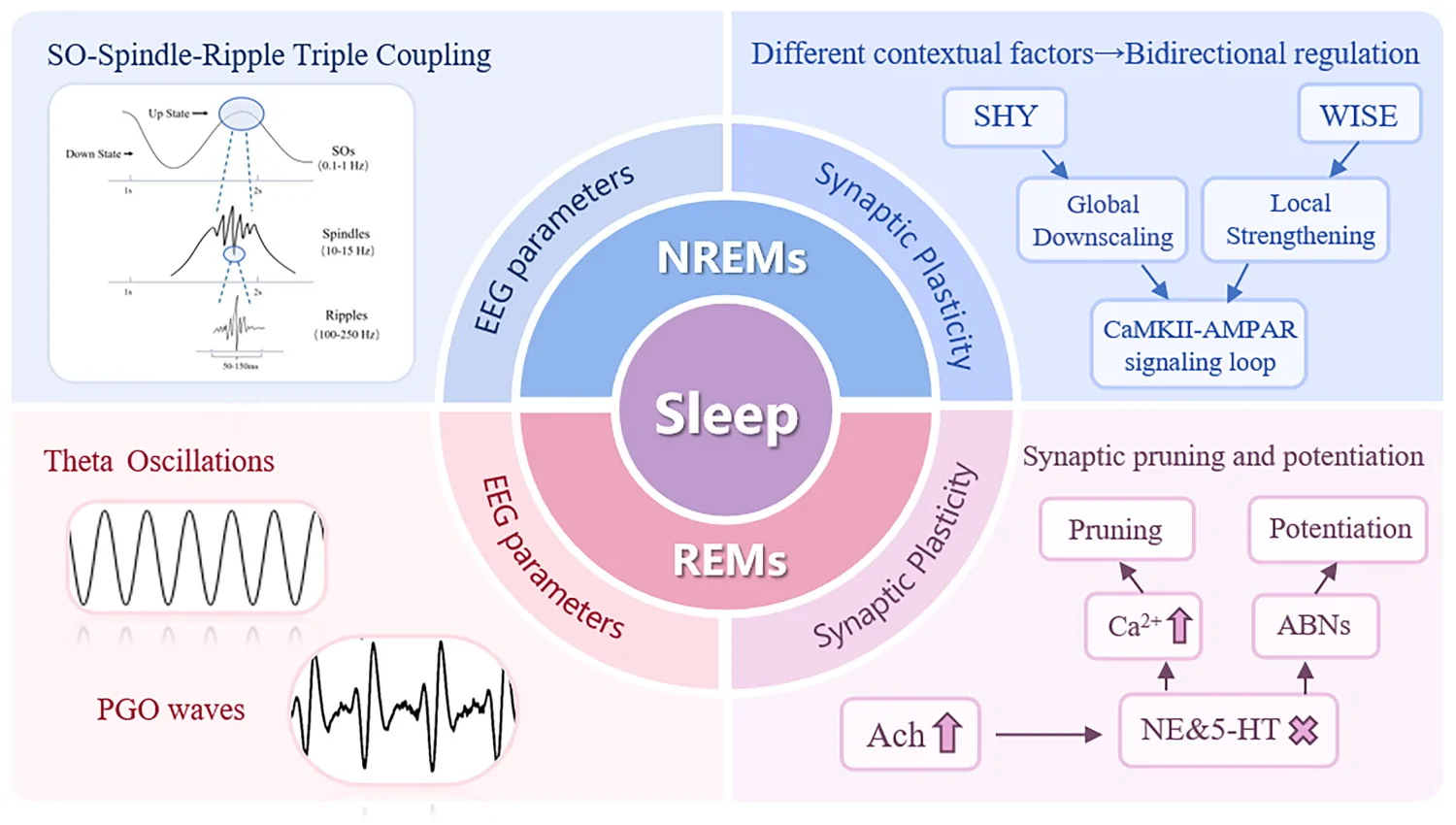
Schematic illustration of bidirectional regulation of synaptic plasticity across sleep. During NREM sleep, EEG features such as slow oscillation-spindle-ripple triple coupling are tightly linked to synaptic plasticity. Depending on contextual factors, the synaptic homeostasis hypothesis (SHY) favours global synaptic downscaling, whereas the WISE framework supports local synaptic strengthening, potentially mediated by CaMKII-AMPAR signalling. In REM sleep, theta oscillations and PGO waves serve as key EEG markers and, together with elevated acetylcholine and changes in norepinephrine and serotonin levels, drive synaptic pruning and potentiation in neuronal populations such as ABNs. These coordinated processes contribute to synaptic remodelling and memory processing during sleep.

## Mechanisms of memory consolidation

During the process of memory encoding, the perception of stimuli leads to the formation of new memory traces. Initially, these traces are highly labile and susceptible to interference and decay-manifesting as forgetting. Memory consolidation refers to the progressive stabilisation of these unstable traces, strengthening the encoded information and integrating it into pre-existing knowledge networks (Marshall and Born 2007).

The concept of memory consolidation, now widely accepted, can be traced back to the seminal work of Müller and Pilzecker in 1900. Based on their studies of retroactive interference in the learning of nonsense syllable lists, they concluded that certain physiological processes support the strengthening of associations formed during initial learning, but that this strengthening diminishes over time (Löwenstein 2008).

The classical consolidation framework encompasses two interrelated levels: **synaptic consolidation** and **systems consolidation**. Synaptic consolidation involves activity-dependent changes in synaptic efficacy that stabilise information storage at the neuronal level. Systems consolidation describes the gradual reorganisation of hippocampus-dependent memories into distributed cortical networks over time. Synaptic consolidation provides the foundation for systems consolidation, while systems-level processes exert reciprocal modulation on synaptic plasticity.

Recent advances have expanded this framework, revealing that following learning, the brain in ‘offline’ states-such as sleep or quiet wakefulness-can produce temporally compressed reactivation of neuronal activity sequences. This phenomenon, termed the **active replay hypothesis**, is thought to drive the transformation of short-term into long-term memory storage (Rubin et al. 2022). Concurrently, evidence suggests that during offline states, a dynamic homeostasis of synaptic strength is maintained: potentiated ‘core’ synapses supporting a memory are selectively reinforced, whereas redundant synapses are weakened, thus refining memory representations.

### Synaptic consolidation

The **Hebbian hypothesis** posits that when two neurons are repeatedly and synchronously activated, the propensity for their coordinated firing becomes durably strengthened (Keysers and Perrett 2004). This phenomenon-known as **long-term potentiation** (LTP)-is considered a fundamental synaptic substrate for the formation of memory traces (Bliss and Gardner-Medwin 1973). In its classic experimental manifestation, stimulation of a presynaptic neuron induces a characteristic excitatory postsynaptic potential (EPSP) in a postsynaptic partner. Upon subsequent stimulation with the same intensity, the amplitude of the EPSP can be markedly increased and this potentiated response may persist from hours to weeks.

The persistence of such synaptic changes underlies the traditional distinction between **short-term memory**-lasting seconds to hours-and **long-term memory**, which may be maintained for days, years, or even a lifetime. Recent evidence suggests that the first wave of synaptic plasticity occurs locally within the hippocampus, conferring contextual specificity to newly acquired information (Redondo and Morris 2011). During sleep on the same day, hippocampal neurons engaged in initial encoding tend to form temporally synchronised assemblies. On the following day, synaptic plasticity within the anterior cingulate cortex becomes essential for further memory stabilization (Takeuchi et al. 2014). These findings underscore that synaptic plasticity exerts distinct, stage-specific roles across different phases of memory consolidation.

### Systems consolidation

**Systems consolidation** refers to the process by which memory representations gradually reorganise from a hippocampus-dependent state to a neocortex-dependent one over time (Frankland and Bontempi 2005; Redondo and Morris 2011) This framework was initially formalised by Squire and Alvarez (1995), who proposed that the hippocampus serves as a temporary repository that directs the gradual strengthening of cortico-cortical connections through repeated coactivation. McClelland and O’Reilly (1995) extended this view through the complementary learning systems theory, emphasising the role of slow neocortical learning in integrating new memories into existing knowledge structures.

Contemporary perspectives have moved beyond a simple hippocampus-to-neocortex transfer model. Frankland and Bontempi (2005) demonstrated that memory reorganisation involves not just transfer but qualitative transformation of representations over time. Dudai et al. (2015) further emphasised that consolidation and reconsolidation represent ongoing, time-dependent processes of reactivation, reweighting and partial reconfiguration. The active systems consolidation framework (Klinzing et al. 2019) specifically highlights how offline states, including sleep, provide optimal conditions for hippocampal replay to drive neocortical integration. More recent work by Golbabaei and Frankland (2025) has updated these theories, incorporating evidence that hippocampal engagement can persist at remote time points and that multiple parallel pathways support different memory types.

### Active replay hypothesis

Memory consolidation is thought to occur primarily during ‘offline’ periods: times when the hippocampus is disengaged from encoding new information-such as during sleep or quiet wakefulness (Klinzing et al. 2019). In these states, the brain actively redistributes recently encoded memories across multiple brain regions through repeated neuronal reactivation (Mednick et al. 2011), integrating them into pre-existing knowledge networks. Initially, this process was presumed to occur exclusively during sleep, leading to the view that sleep was indispensable for consolidation (Wamsley 2019). However, accumulating evidence demonstrates that similar active neural replay also takes place during restful wakefulness (Dastgheib et al. 2022).

Both sleep- and wake-associated replay have been shown to strengthen synaptic connections within memory traces, thereby enhancing their stability (Buzsáki 1996; Dastgheib et al. 2022). Advances in computational neuroscience and neuroimaging now permit the detection of replay-like activity in humans using non-invasive methods, enabling direct investigation of these processes *in vivo* (Buzsáki 2015; Wittkuhn and Schuck 2021).

### Models of memory consolidation

Theoretical frameworks of memory consolidation have undergone substantial revision since the concept was first introduced in the early twentieth century.

#### Classical/standard consolidation model

The Standard Consolidation Model was proposed in the 1990s (Klinzing et al. 2019), it also referred to as the classical consolidation model. The model posits that newly acquired memories are initially stored in the hippocampus and, over time, are transferred to the neocortex for long-term storage (Kitamura and Inokuchi 2014; Liu et al. 2021). In this framework, the hippocampus functions as a temporary repository, while the neocortex serves as the permanent storage site. Early in this process, the distributed memory traces within the neocortex are only weakly interconnected. Through repeated coactivation by corresponding hippocampal memory circuits, these connections are progressively strengthened. Once robust cortico-cortical connections are established, hippocampal representations decay and memory retrieval no longer depends on the hippocampus (Frankland and Bontempi 2005).

Because both the hippocampus and the neocortex participate in encoding new information, the transfer and integration of memories are thought to occur predominantly during offline states-periods when external sensory input is minimal-such as during sleep^1^ or quiet wakefulness (Dastgheib et al. 2022).

#### Multiple trace theory

With the accumulation of empirical evidence, it became clear that the Standard Consolidation Model had certain limitations. Nadel et al. proposed the Multiple Trace Theory, which supplements this part very well (Nadel and Moscovitch 2001). Multiple Trace Theory (MTT) proposes that traces in the hippocampus are formed rapidly and obligatorily encode all episodic information. Acting as an ‘index’ to memory traces in the neocortex, the hippocampal trace binds these cortical elements into a coherent memory circuit. Hippocampal connections to perceptual and spatial cortical systems contribute fine-grained local details, whereas connections to conceptual cortical systems provide global contextual frameworks (Liu et al. 2016). The consolidation of hippocampally stored short-term memories is selective and subject to modulation by factors such as cues, reward, emotion and pre-existing schemas. Furthermore, the transfer process can no longer be adequately characterised as a single hippocampus-to-neocortex route; instead, different memory types and distinct cortical territories appear to be supported by multiple, partially parallel pathways. Hippocampal-cortical interactions can also persist at remote time points, and retrieval of long-term memories that are primarily neocortically based can still elicit hippocampal activity (Takeuchi et al. 2014).

A refined version of MTT emphasises dissociable roles of cortical and hippocampal storage: cortical representations are critical for generalised semantic memory, whereas hippocampal traces preserve detailed episodic content (Golbabaei and Frankland 2025).

#### Integrated cortico-Amygdalo-Hippocampal model

In a landmark study published in the journal Science in 2017 (Kitamura et al. 2017), Kitamura and his colleagues proposed an updated framework for their research on memory trace cells. It was discovered that the consolidation of episodic fear memory at the cellular level begins at the moment of encoding. By integrating cell labelling techniques, AAV-based transgenic mouse models, circuit tracing, optogenetics and calcium imaging, the authors investigated the role of imprinted cells and circuits spanning the medial prefrontal cortex (PFC) and subcortical regions, including the medial entorhinal cortex (MEC), the hippocampus and the basolateral amygdala (BLA). They found that during the process of memory formation, the hippocampus promotes the rapid generation of immature imprinted cells in PFC. During the system consolidation process, the hippocamp-MEC-PFC and BLA-PFC pathways act together to mature these PFC-imprinted cells. During retrieval, behavioural expression can be driven by hippocampal-amygdala interaction without the need for hippocampal-neocortex transfer. This model redefines PFC as a key site for the formation and stability of long-term memory.

Subsequent studies found that when long-term situational fear memories are retrieved again after they have completed ‘system consolidation’ and are behaviourally dependent on neocortex (mPFC), the hippocampus will be rerecruited (Klinzing et al. 2019). This process is called system reconsolidation. Essentially, it is a process of ‘reconstructing new hippocampal memory traces’. This new engram rebinds the original, already cortical memory content with the newly obtained contextual information at present, thereby achieving the update of long-term memory and even providing the cellular and circuit basis for the formation of ‘false memory’.

Hamed et al. (Kitamura and Inokuchi 2014) further conducted research from the perspective of reward memory and systematically examined the spatiotemporal dynamic changes from recent to remote recall in multiple brain regions. They found that during long-term extraction, overall activity declined but the ‘specialised neural subgroups’ were more stable, and memory consolidation relied less on more specialised cell populations. Memory retrieval is highly dependent on motivation and individual differences.

Table 1 summarises how major nodes of the hippocampal-cortical-emotion/reward circuitry contribute to encoding, retrieval, systems consolidation and remote expression of episodic memories. For each region, we distinguish a local ‘minimal core function’ (e.g., pattern separation in dentate gyrus, pattern completion in CA3, output integration in CA1, abstraction in mPFC, dopaminergic tagging in VTA, emotional valence in amygdala) and a ‘minimal network role’ within the larger circuit This compact framework highlights the division of labour and coordinated interactions that support the formation, stabilisation and updating of episodic memory engrams.

**Table 1.** Key memory-related brain regions, their predominant stages of involvement and their minimal core functions and network roles in episodic memory.

Brain region	Main stage(s) (abbr.)	Minimal core function	Minimal network role
Dentate gyrus (DG)	Encoding, early retrieval	Entry gate for episodic memory, strong pattern separation, can be rewritten during reconsolidation	Sends highly separated inputs into the hippocampal network, allowing remote memories to be updated when they are retrieved (Carretero-Guillén et al. 2024)
CA3	Encoding, early retrieval	Pattern completion: reconstructs full episodes from partial cues	Rebuilds memories based on DG activity and sends the integrated episodic pattern to CA1/cortex (Vancura et al. 2023)
CA1	Encoding, recent/intermediate retrieval	Main hippocampal output hub, maintains core of recent and some remote memories	Links hippocampus with EC/neocortex and forms a temporally persistent ‘core’ of the engram (Bai et al. 2023)
Entorhinal cortex (EC)	Encoding, early systems consolidation	Source of spatial + contextual information, early ‘proto-cortical’ engram	Acts as the input/output gate between hippocampus and cortex, conveying a ‘spatial scaffold + event details’ to cortical networks (Zhang et al. 2023)
Medial PFC (mPFC)	Mid/late consolidation, remote retrieval	Center for remote, abstract and value-based memories, flexibly updatable	Long-term store for engrams transferred from hippocampus; dominates remote memory expression and reintegration (Khan et al. 2024)
Amygdala	All stages (emotional)	Assigns positive/negative emotional valence and scales memory strength	Adds emotional weight to hippocampal/cortical memories and maintains long-term approach/avoidance biases (Saito et al. 2025)
Ventral tegmental area (VTA)	Encoding, consolidation modulation	Dopamine ‘tagger’ for long-term storage/updating	Decides which engrams are consolidated or updated, providing motivational signals for systems (re)consolidation (Glykos and Fujisawa 2024)
Nucleus accumbens (NAcc)	Expression, habit	Converts context + value into reward-seeking/habitual actions	Integrates inputs from VTA, hippocampus, mPFC and amygdala to generate approach/avoidance decision biases (Hamed et al. 2025)
Other cortex	Mid/late consolidation, remote detail	Distributed engrams for concrete sensory and spatial details	Complements mPFC’s abstract representations and the hippocampal scaffold to form the long-term memory network (Said and Miyamoto 2025)

## Memory consolidation during sleep

Polysomnographic recordings-based on electroencephalography (EEG), electromyography (EMG) and electrooculography (EOG)-classify sleep into rapid eye movement sleep (REM sleep) and non-rapid eye movement sleep (NREM sleep), which alternate cyclically throughout the night. NREM sleep is further subdivided into three stages (N1, N2, N3) of increasing depth, with N3-also called slow-wave sleep (SWS)-representing the deepest stage. SWS and REM are the two principal sleep states in mammals: in humans, SWS predominates early in the night and declines in intensity and duration across successive cycles, whereas REM becomes more intense and frequent towards the end of the sleep period (Rasch and Born 2013) (Figure 2).

**Figure 2.**
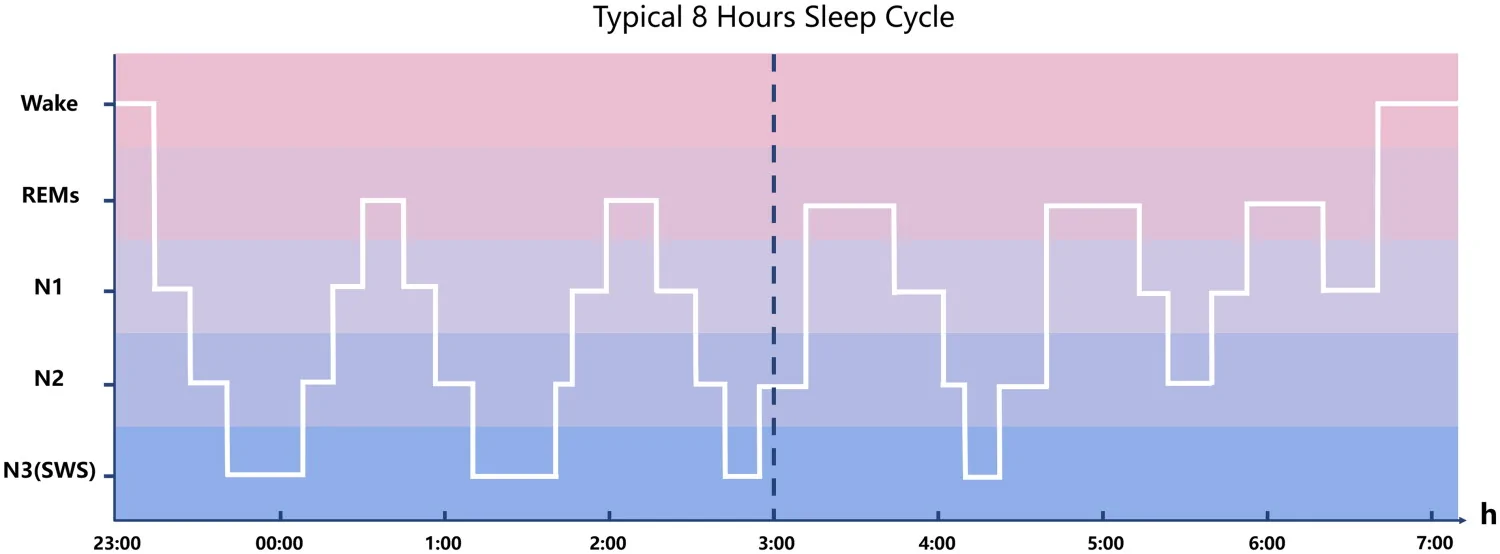
Schematic representation of the human sleep architecture. The white step line depicts the progression of sleep stages across the night, cycling from wakefulness through N1, N2, N3 to REM sleep in recurring 90–120 min ultradian cycles. The coloured background illustrates the transition from deep to lighter NREM sleep and REM, while the dashed vertical line marks the midpoint of the night, highlighting the predominance of N3 in early sleep and of REM and lighter stages in late sleep.

Early research on the sleep-memory relationship relied heavily on sleep deprivation paradigms. By the mid-twentieth century, it was demonstrated that depriving subjects of sleep after training impairs memory retention, whereas post-learning sleep improves performance, providing direct evidence for the role of sleep in memory consolidation. Subsequent behavioural studies-using classical, aversive and appetitive conditioning paradigms-have shown consistent increases in REM sleep following learning in rats, mice and cats. Environmental enrichment also enhances post-learning REM duration, although increases in NREM are also observed (Rasch and Born 2013).

In a classic shuttle-box avoidance study, selective REM deprivation immediately after training abolished improvements in task performance. Similar patterns have been observed in both hippocampus-dependent (e.g., Morris water maze, contextual fear conditioning) and hippocampus-independent tasks (e.g., complex operant conditioning) (Maquet 2001). That enriched environments or learning experiences alter subsequent sleep architecture indirectly supports the notion that consolidation depends on sleep. Prolonged exposure of rats to enriched environments for 3 weeks significantly lengthens subsequent sleep duration. In humans, second-language learning has been shown to produce proportional increases in total sleep time, which strongly correlate with recall performance (Xia et al. 2023).

With the advent of modern neuroscience techniques, research has moved from macroscopic behavioural observations to the microscale, examining neuronal firing patterns, synaptic morphology and molecular processes. As discussed earlier, mechanisms such as synaptic homeostasis are thought to be sleep-dependent, while active replay occurs predominantly during sleep. In the sections that follow, we examine how distinct stages of sleep contribute to memory consolidation through these mechanisms.

### Associated neurophysiological activity during NREM sleep

In healthy adults, the electroencephalogram (EEG) during slow-wave sleep (SWS) is dominated by three canonical oscillatory patterns: slow oscillations (SOs; 0.1-1 Hz) originating in the neocortex (Timofeev et al. 2000), sleep spindles (10–15 Hz) generated by the thalamic reticular nucleus (Antony et al. 2018) and sharp-wave ripples (SWRs; 100–250 Hz) arising in the hippocampus (Csicsvari et al. 2000). These neural oscillations reflect rich spatiotemporal dynamics within the sleeping brain and can be selectively synchronised across brain regions, thereby facilitating interregional communication, informationtransfer and the transformation of transient memories into long-term storage (Helfrich et al. 2021).

#### Slow oscillations: temporal windows for memory consolidation

During non-rapid eye movement (NREM) sleep, slow oscillations (SOs) reflect rhythmic fluctuations in the membrane potential of neuronal assemblies, alternating between depolarised UP states and hyperpolarized DOWN states. UP states are marked by synchronous bursts of excitatory and inhibitory synaptic activity, whereas DOWN states correspond to global neuronal quiescence (Vyazovskiy et al. 2009). SOs are initiated in layer 5 pyramidal neurons (Sanchez-Vives and McCormick 2000) and propagate to layer 2/3 (Chauvette et al. 2010), with both layers contributing to their generation and horizontal propagation. These oscillations propagate as travelling waves through thalamo-cortical loops, bidirectionally engaging subcortical structures such as the hippocampus. This large-scale propagation imposes phase alignment of activity across distributed regions and thereby furnishes a spatiotemporal scaffold for systems consolidation (Muller et al. 2018).

Across the literature, three complementary levels of analysis-interventional, network-level and cellular-converge on a mechanistic role of SOs, while clarifying that their effects are strongly phase-dependent. At the interventional level, Marshall et al. first showed that enhancing SO activity *via* slow-oscillatory transcranial direct current stimulation (tDCS) during NREM sleep improves subsequent memory, arguing against a purely epiphenomenal status of SOs (Marshall et al. 2006). Later work using rhythmic electrical, magnetic and auditory stimulation refined this view, demonstrating that hippocampal ripples and sleep spindles support memory transfer most effectively when phase-locked to the SO UP state (Marshall et al. 2006; Massimini et al. 2007; Vyazovskiy et al. 2009). Within this line, phase-locked auditory paradigms by Ngo and colleagues revealed that stimulation during the rising phase of SOs amplifies their amplitude and duration and strengthens SO-spindle coupling, leading to superior declarative memory retention (Ngo et al. 2013). Direct comparisons of UP- versus DOWN-state cueing further showed that only UP-state stimulation reliably reduces forgetting and boosts spindle power, underscoring a functional asymmetry between SO phases (Ngo and Staresina 2022). Clinically, Weinhold et al. reported reduced UP-state spindle power in schizophrenia, alongside memory deficits, suggesting that impaired SO-spindle coupling-rather than reduced SO incidence alone-may underlie cognitive symptoms (Weinhold et al. 2022).

Network-level analyses characterise how SOs structure large-scale communication. Using causal information-flow metrics, Niknazar et al. found that global connectivity is generally suppressed during NREM sleep by around 40%, but transiently increases by roughly 78% during SO UP states (Weinhold et al. 2022). When combined with stimulation studies, this pattern supports the idea that SOs periodically open and close ‘communication windows’ during which hippocampo-neocortical exchanges and memory reactivation are preferentially expressed.

At the cellular scale, Mittermaier et al. used patch-clamp recordings from resected human neocortical tissue to show that subthreshold UP/DOWN oscillations modulate axonal ion channels, enhance synaptic transmission and facilitate associative plasticity during slow-wave sleep (Mittermaier et al. 2024). These findings provide a mechanistic link to the network and behavioural data: during UP states, neurons are biased towards firing in response to spindle- and ripple-related inputs, promoting synaptic strengthening of active assemblies, whereas DOWN states transiently suppress background activity and may improve the signal-to-noise ratio of reactivated traces.

Finally, closed-loop targeted memory reactivation (CL-TMR) studies indicate that these principles extend beyond declarative memory. Nicolas et al. showed that UP-state-locked CL-TMR of motor sequences selectively increased SO amplitude and density, strengthened striato-motor cortical network activity and preserved hippocampal engagement (Nicolas et al. 2025). Together with declarative findings, this suggests that SO UP-state gating constitutes a domain-general mechanism through which distributed networks are synchronously recruited to support consolidation.

In sum, these studies collectively move the field beyond a ‘more SOs is better’ view towards a phase- and coupling-based framework: SOs periodically organise large-scale communication, align spindles and ripples into optimal temporal windows, and modulate synaptic plasticity, with the contrast between UP and DOWN states-and their coupling to faster rhythms-emerging as the critical determinant of memory outcomes.

#### Sharp-wave ripples: hippocampal reactivation of memory traces

Hippocampal sharp-wave ripples are large-amplitude, irregular local field potentials composed of high-frequency ripple oscillations (100–250 Hz) superimposed on low-frequency sharp waves (0.01-3 Hz) (Ngo et al. 2013). Their generation involves a three-stage circuit mechanism: (1) spontaneous synchronous bursting of CA3 pyramidal neurons generates sharp waves that depolarise CA1 apical dendrites *via* Schaffer collateral inputs (Ngo and Staresina 2022); (2) parvalbumin-positive (PV^+^) interneurons in CA1 organise these depolarizations into high-frequency ripples, which are further amplified by CA3-CA1 feedback loops (Weinhold et al. 2022); and (3) inhibitory control from CA2 interneurons suppresses CA3 activity, functioning as a gating ‘switch’ for SWR initiation (Buzsáki 2020).

At the single-event level, SWRs provide a temporal scaffold for compressed neuronal replay (Staresina et al. 2015). In rodents, closed-loop disruption of SWRs during post-training sleep impairs coordinated reactivation of hippocampal ensembles and produces pronounced spatial memory deficits, demonstrating that SWRs are necessary for effective off-line reexpression of experience (Mittermaier et al. 2024). In humans, intracranial recordings show that hippocampal populations reinstate waking sequences in temporally compressed form during NREM-related ripples, and that the strength and precision of this replay predict next-day memory performance, indicating that SWR-associated replay is a conserved mechanism of episodic memory stabilization (Nicolas et al. 2025).

Beyond the hippocampus, SWRs are embedded in recurrent cortico-hippocampal-thalamo loops (Grosmark et al. 2021). Simultaneous recordings from neocortex and hippocampus reveal bidirectional cortico-hippocampo-cortical communication during SWRs, with cortical activity both triggering hippocampal ripples and being reshaped by ripple-linked replay (Adamantidis et al. 2019; Girardeau and Lopes-dos-Santos 2021). Causal manipulations highlight that it is not simply the occurrence, but the context and patterning of SWRs that matter: prolonging ripples that arise in behaviourally relevant states selectively enhances memory, whereas randomly extending SWRs has no effect, emphasising that only ‘content-appropriate’ ripples contribute to consolidation (Oliva et al. 2016). High-resolution analyses further show that SWRs are most effective when nested within specific phases of slow oscillations and spindles (Logothetis et al. 2012; Schreiner et al. 2024), suggesting that precise cross-frequency coupling gates when ripple-driven replay can influence cortical plasticity.

At the circuit scale, learning-dependent changes in hippocampal and cortical connectivity determine which memories are replayed (Oliva et al. 2016; Said and Miyamoto 2025). Data-driven modelling of CA3 recurrent networks indicates that synaptic structures formed during learning both bias reactivation content and are essential for SWR generation, directly linking engram-like connectivity to ripple dynamics (Girardeau et al. 2009). In parallel, cell-type-specific imaging demonstrates that thalamocortical silencing in the seconds preceding SWRs reduces distal dendritic inhibition of pyramidal neurons, thereby optimising slow-oscillation-ripple alignment and selectively facilitating LTP at synapses carrying memory-relevant information (Zhang et al. 2018). These findings imply that subcortical and cortical circuits proactively shape the excitatory-inhibitory landscape into which hippocampal replay arrives.

Recent work also broadens the cortical partners involved in SWR-associated consolidation. The retrosplenial cortex (RSC), for example, shows pre-SWR activation of excitatory neurons and post-SWR engagement of inhibitory neurons during spatial tasks, a pattern consistent with RSC providing preparatory input to hippocampal replay and then stabilising local cortical activity (Rothschild et al. 2017; Karimi Abadchi et al. 2020). Such results support a view of consolidation as a distributed computation coordinated across multiple cortical territories (Giri et al. 2024).

The state dependence of SWR function underscores that quality of replay is crucial. Although hippocampal replay also occurs during quiet wakefulness, sleep deprivation distorts SWR physiology and severely weakens their reactivation efficacy (Fernández-Ruiz et al. 2019). Critically, these impairments persist despite recovery sleep, suggesting that there is a limited temporal window in which high-fidelity SWR-linked replay must occur for successful consolidation (Fernández-Ruiz et al. 2019).

Collectively, these findings indicate that hippocampal SWRs, embedded within precise cortico-hippocampal-thalamo interaction patterns and sculpted by cell-type-specific inhibitory control, transform unstable hippocampal traces into distributed, durable cortical memories (Staresina et al. 2015).

#### Thalamic spindles in the modulation of systems consolidation

Sleep spindles-oscillatory events in the 10–15 Hz range-are generated during NREM sleep by the thalamic reticular nucleus (TRN) and shaped by thalamo-cortical interactions before propagating *via* thalamocortical projections to the neocortex and hippocampus (Fernandez and Lüthi 2020).

Converging human and animal evidence indicates that sleep spindles and associated thalamo-cortical dynamics are not merely epiphenomena of NREM sleep, but mechanistically support memory consolidation. In humans, Mak-McCully et al. showed that cortical SO DOWN states hyperpolarize thalamic neurons *via* cortico-thalamic projections, reliably triggering spindle bursts that are relayed back to cortex during the DOWN-UP transition, thus embedding spindles within a hierarchical SO framework that can gate plasticity-relevant communication between distributed areas (Mak-McCully et al. 2017). Dickey et al. extended this network view using intracranial recordings, demonstrating that thalamic ripples occur largely independently of cortical and hippocampal ripples, while SO UP states and spindles temporally coordinate cortico-hippocampal co-ripples, pointing to a role for spindles as integrative carriers that synchronise local high-frequency replay across structures (Dickey et al. 2024). At the systems level, scalp-EEG and polysomnographic work has shown that the spatial distribution and timing of spindle activity preferentially align with cortical territories recruited during prior learning, and that this spatial-temporal concordance predicts subsequent behavioural gains, consistent with a ‘targeted systems consolidation’ framework in which spindles selectively stabilise recently engaged representations (Cairney et al. 2018; Petzka et al. 2022; Sjøgård et al. 2025). Complementing these human data, rodent work indicates that strengthening associative memories is accompanied by enhanced engagement of integrative network hubs during NREM sleep (Wang et al. 2023). Collectively, these findings suggest a functional taxonomy in which: (1) SOs provide global windows for plasticity (Mak-McCully et al. 2017); (2) thalamo-cortical spindle bursts implement selective routeing and gain control within those windows (Barthó et al. 2014; Cairney et al. 2018; Petzka et al. 2022; Sjøgård et al. 2025); and (3) locally generated ripples support content-specific replay that is bound into coherent memory traces through spindle-mediated cross-regional coupling (Dickey et al. 2024). Within this scheme, differences across studies in task demands, recording level (scalp vs. intracranial), and targeted structures can be understood as emphasising distinct nodes or temporal phases of a common SO-spindle-ripple consolidation circuit.

Phase-amplitude coupling during NREM sleep emerges as a structured yet functionally heterogeneous mechanism rather than a unitary ‘consolidation signal’. Across EEG, EEG-fMRI and intracranial work, slow oscillation (SO) UP states reliably phase-lock thalamocortical spindles, whose high-frequency components at UP-state peaks gate dendritic Ca^2+^ influx and local plasticity in neocortical circuits, thereby optimising conditions for synaptic change rather than globally boosting excitability alone (Seibt et al. 2017). However, only a subset of these events-those precisely nested within stereotyped SO-spindle complexes-appear to support systems-level memory processing. Hippocampal sharp-wave ripples (SWRs) preferentially lock to spindle troughs, forming spindle-SWR clusters that synchronise CA1 place-cell sequences with target neocortical sites and enable directed transfer of memory traces (Antony et al. 2019). Optogenetic interventions further reveal that disrupting SO-spindle coupling during the UP state impairs consolidation, whereas disrupting δ waves promotes forgetting, indicating a causal rather than merely correlative role and revealing that SOs and δ waves serve competing functions in sleep-dependent memory processing (Kim et al. 2019). Multivariate decoding studies show that endogenous memory reactivation co-occurs specifically with nested SO-spindle coupling and that coupling precision, rather than spindle density, tracks reactivation strength and behavioural consolidation (Schreiner et al. 2021; Cross et al. 2025). Converging EEG-fMRI evidence demonstrates that SO-spindle coupling preferentially drives hippocampal reactivation and simultaneously recruits frontal regions to facilitate hippocampo-neocortical exchange (Baena et al. 2022). Combined scalp/intracranial EEG with targeted memory reactivation (TMR) reveals that only spindle-locked medial temporal ripples carry decodable mnemonic content, underscoring hierarchical SO-spindle-ripple coupling-not isolated oscillations-as a core, mechanistically specific motif of systems consolidation (Schreiner et al. 2024).

Recent work suggests that sleep spindles influence hippocampal activity primarily through multi-synaptic thalamo-cortico-hippocampal loops rather than *via* a single direct pathway. In humans, recordings from the anterior thalamic nuclei (ATN) show that local slow-oscillation (SO) peaks and spindles systematically precede cortical SOs by tens of milliseconds (Schreiner et al. 2022), indicating that anterior thalamus can impose a temporal scaffold on widespread cortical targets. Complementing this systems-level view, intracranial studies of individual differences in memory consolidation have linked stronger SO–spindle–ripple coupling to more robust overnight retention, suggesting that the effectiveness of these loops is a critical determinant of consolidation success (Duan et al. 2025). In mice, optogenetic and imaging data further identify the anteromedial thalamus (AM) as a limbic hub that gates which memories are routed into thalamo-cortical loops for long-term stabilization (Toader et al. 2023). Given the dense projections from anterior thalamic nuclei to medial prefrontal and retrosplenial cortices, and from there to entorhinal and parahippocampal regions, these findings support a largely trisynaptic route: anterior-thalamic spindle volleys first entrain limbic cortices, which then drive hippocampal subfields *via* entorhinal inputs to dentate gyrus/CA3 and CA1.

Physiologically, rodent recordings show that long-duration, low-frequency anterior thalamic spindles selectively increase hippocampo-thalamic synchrony and sharpen spindle–ripple coupling (Alizadeh et al. 2022), in line with a recurrent loop in which thalamic input not only paces, but also gates, the timing of hippocampal sharp-wave ripples. Human depth recordings across hippocampal subfields reveal a layered oscillatory organisation: dentate gyrus/CA3 provide broad delta–ripple coordination, whereas CA1 and subiculum express precise delta–spindle–ripple nesting (Li et al. 2024). This laminar pattern is compatible with stronger cortical and entorhinal drive onto CA1/subiculum, which then project back to thalamus and frontal cortex, closing the loop. These anatomical and electrophysiological data imply that anterior thalamic spindles reach the hippocampus predominantly *via* limbic-cortical and entorhinal relays, embedded in closed thalamo-hippocampo-cortical circuits that allow spindles to both pace and selectively stabilise hippocampal replay (Figure 3).

**Figure 3.**
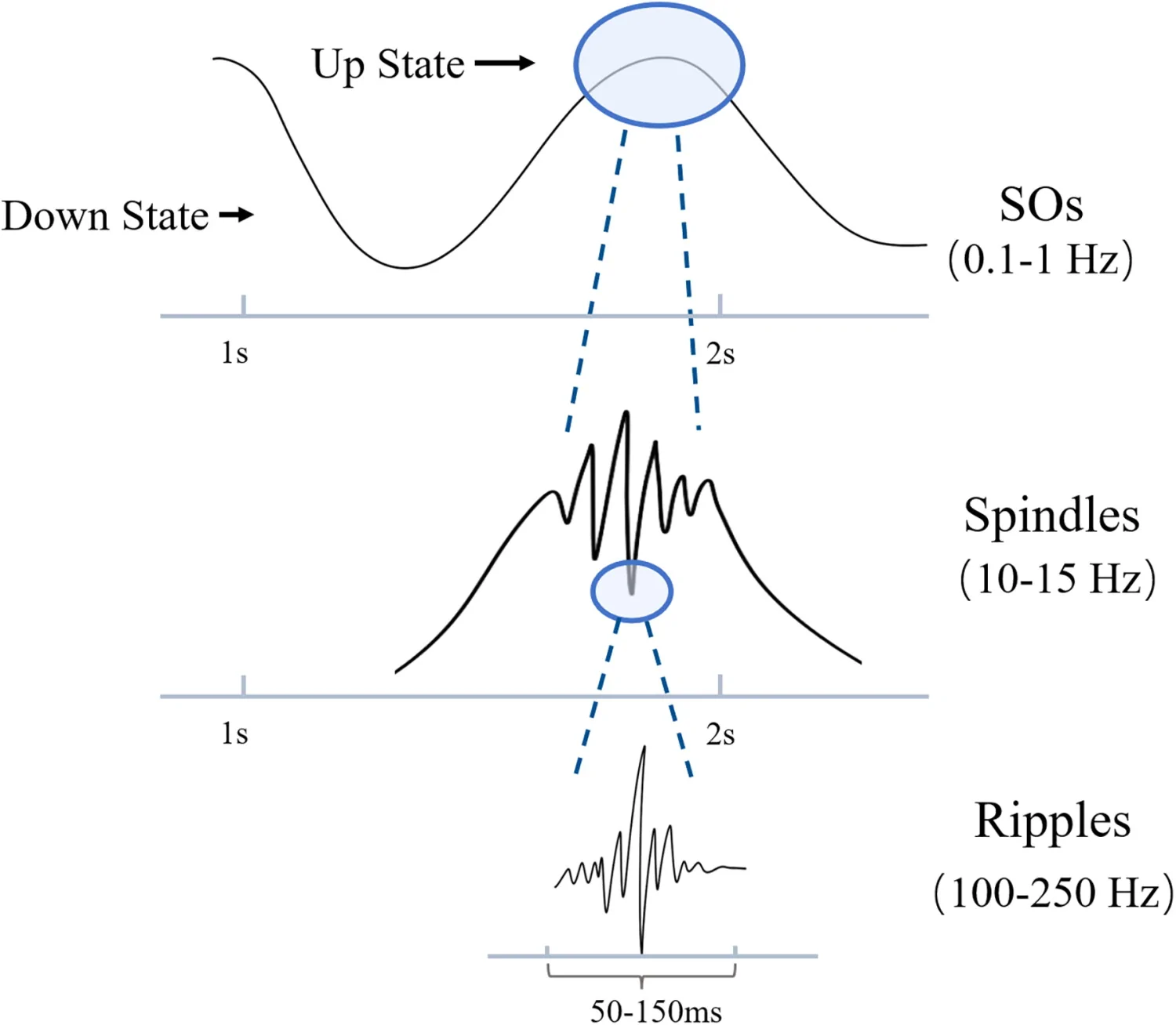
Schematic representation of NREM sleep oscillations based on local field potential (LFP) recordings: (1) Cortical SOs provide a cyclical temporal framework (UP/DOWN states) for information transfer and synaptic plasticity. (2) SO UP states trigger thalamo-cortical spindle activity; precise SO-spindle phase coupling ensures that this thalamic-cortical synchrony aligns with hippocampal reactivation. (3) Hippocampal SWRs emerging during reactivation are nested within spindle troughs, forming integrated spindle-SWR events that synchronise hippocampal and neocortical ensembles. The neocortex then engages in synaptic consolidation, embedding the transferred trace into long-term cortical storage.

### Other NREM-related changes relevant to memory consolidation

#### Synaptic modifications during NREM sleep

Tononi and Cirelli first proposed the synaptic homeostasis hypothesis (SHY) (Tononi and Cirelli 2003), suggesting that during wakefulness, long-term potentiation (LTP) strengthens specific synapses to form memory traces, which may, however, lead to excessive potentiation of network activity. Therefore, the global synaptic downscaling occurring during NREMs weakens non-critical connections, thereby restoring the balance of network excitability while preserving the relative strength differences established by LTP. This homeostatic regulation of synaptic strength not only prevents synaptic saturation and avoids ‘catastrophic interference’, but also optimises energy consumption and the signal-to-noise ratio of memories (Sara 2017).

Vyazovskiy et al. provided the first molecular and electrophysiological evidence for alternating net synaptic potentiation during wakefulness and net synaptic depression during sleep, independent of circadian phase-cortical SO amplitude decreases predicted the magnitude of synaptic weakening (Vyazovskiy et al. 2008). Diering et al. found that adenosine-driven recruitment of Homer1a to excitatory synapses during sleep enables Homer1a-dependent scaling, crucial for discrimination of fear memories (Diering et al. 2017). Direct ultrastructural evidence from de Vivo et al. showed that ∼80% of cortical synapses shrink during sleep, supporting SHY (de Vivo et al. 2017). Subsequent work by Norimoto et al. identified hippocampal SWRs during NREMs as a neural orchestrator that actively and selectively weakens non-critical connections *via* NMDAR-dependent synaptic depression, thereby downscaling synaptic weights and providing key physiological evidence for the SHY (Norimoto et al. 2018). The hypothesis was further refined by Suppermpool et al. who demonstrated that sleep pressure in zebrafish dynamically regulates synapse number at the single-neuron level-increasing during wakefulness and decreasing under high sleep pressure-in a cell-subtype-specific manner, a process requiring adenosine signalling and low noradrenergic tone (Lyons et al. 2024).

Memory reactivation, which remodels hippocampal traces by potentiating synaptic transmission or altering synaptic weights, is a key cellular mechanism underpinning memory consolidation during NREMs (Zhou and Norimoto 2023). Experimental evidence indicates that NREM sleep critically mediates synaptic weakening, with one study showing that 5 h of recovery sleep suffice to reverse sleep deprivation-induced synaptic changes, even in ageing models (Squarcio et al. 2024). Furthermore, slow-wave sleep (SWS) has been identified as a core driver of AMPA receptor renormalisation in the hypothalamus, extending the synaptic homeostasis hypothesis beyond memory circuits to central homeostatic regions, a process potentially supported by thalamic spindles that preserve critical synapses (Liu et al. 2024).

Subsequent research, however, has reported observations of increased synaptic strength during NREMs, which appear inconsistent with the predictions of the SHY.

For example, in visual cortex, sleep selectively strengthens non-deprived eye pathways *via* NMDAR-PKA signalling, paralleling the reactivation-NMDAR cascade implicated in memory (Aton et al. 2009). Chauvette et al. observed that hyperpolarized silent states in SWS trigger LTP (Chauvette et al. 2012), and Sadowski et al. directly showed that SWR-driven replay induces STDP-like plasticity (Sadowski et al. 2016). Raven proposed a nuanced view: sleep supports consolidation mainly by preserving and reinforcing necessary structural connections, rather than simply downscaling (Raven et al. 2018). Xu et al. argued that sleep restores cortical network ‘criticality’, optimising computational capacity (Xu et al. 2024). Unique neurochemical states during sleep may further promote adaptive overnight emotional memory changes (Cabrera et al. 2024). Advanced synaptome mapping by Koukaroudes et al. revealed that sleep maintains excitatory synaptic diversity, especially in cortex and hippocampus, and that sleep loss rapidly reduces diversity, disproportionately affecting short-lived protein subtypes (Koukaroudi et al. 2024).

Alternative frameworks, such as the synaptic renormalisation theory, suggest that sleep calibrates rather than indiscriminately weakens synapses: Watson et al. showed NREM reduces firing-rate disparities, REM provides global inhibition and microarousals selectively boost low-rate neuron activity (Watson et al. 2016). Crelli et al. confirmed structural miniaturisation of synapses varies by brain region and waking state, suggesting region- and state-specific plasticity (Cirelli and Tononi 2020). Marchal et al. modelled slow (4–7 h) oscillations of synaptic strength across hippocampal circuits, with wake enhancing and both SWS and REM reducing strength (Marchal et al. 2025).

Most recently, Fukuaki et al. developed a unifying computational model integrating Hebbian/STDP rules with the WISE mechanism (synchronous depolarizations in NREM increasing synaptic efficacy *via* Ca^2+^ influx) and the SHY mechanism (Anti-Hebbian suppression). This dual-framework reconciles the apparent dichotomy between enhancement and downscaling, suggesting that the balance is determined by boundary conditions and underlies NREM’s dual role in optimising signal-to-noise ratios while restructuring circuits *via* CaMKII-AMPAR feedback loops (Kinoshita et al. 2025).

#### Neuromodulatory and physiological orchestration of NREM sleep

The diverse synaptic outcomes described above—ranging from selective weakening to pathway-specific strengthening—suggest the need for overarching modulatory frameworks that coordinate when and where plasticity occurs during NREM sleep. Poe et al. (2020) synthesise prior work showing that state-dependent NA release biases memory consolidation or weakening across NREM and REM sleep. Extending this, Kjaerby et al. (2022) demonstrate that infra-slow NA oscillations during NREM create distinct substates where fluctuation amplitude predicts spindle density and memory performance. A recent review (Sulaman et al. 2024) further contextualises LC–NA within broader neuromodulatory interactions underlying NREM-dependent memory processing. Collectively, these studies shift the field from a linear arousal model to a dynamic framework where the temporal structure of LC–NA activity critically determines which memories are stabilised or forgotten. This framework provides a unifying lens through which to view a growing body of work identifying specific physiological signatures of NREM sleep that are functionally linked to memory consolidation.

Consistent with this dynamic neuromodulatory view, recent studies have begun to dissect the fine-grained temporal structure of NREM sleep. Using pupillometry as a peripheral readout of LC activity, Chang et al. (2025) delivered optogenetic stimulation time-locked to sharp-wave ripple (SWR) detection in freely moving mice, while simultaneously tracking pupil diameter. They discovered that NREM sleep exhibits slow cyclical oscillations of pupil constriction and dilation. These micro-substates were differentially associated with memory consolidation: the constricted phase preferentially replayed newly acquired memories, whereas the dilated phase favoured replay of remote memories. Critically, selective disruption of neural activity only during the pupil-constricted ‘new memory replay’ phase impaired consolidation, whereas interference in the dilated phase had no effect. This provides causal evidence that the brain temporally segregates replay of recent and remote memories during sleep to minimise interference, likely under noradrenergic control.

Probing downstream effectors of noradrenergic signalling, Deng et al. (2025) employed high-temporal-resolution *in vivo* cAMP imaging to reveal, for the first time, ultra-slow oscillations (∼1 min cycle) of cAMP levels across multiple brain regions, including hippocampus and prefrontal cortex, during NREM sleep. These oscillations were highly synchronised and critically dependent on β_1_-adrenergic receptor signalling. Using closed-loop optogenetics, the authors demonstrated that hippocampal activity was essential for memory consolidation only at the peak of the cAMP oscillation; selective suppression of hippocampal firing during this peak window markedly impaired spatial memory. This identifies an intrinsic cAMP-dependent ‘pro-plasticity time window’ within NREM sleep, during which hippocampo-cortical functional connectivity is transiently strengthened—a direct mechanistic link between LC-NA activity and optimal conditions for memory stabilisation.

Extending the neuromodulatory framework to incorporate bodily rhythms, Schwimmbeck et al. (2025) propose that respiration orchestrates systems memory consolidation during NREM sleep by acting as a physiological pacemaker. The respiratory rhythm modulates and temporally coordinates slow oscillations, spindles and hippocampal sharp-wave ripples. This synchronisation facilitates hippocampo-cortical communication, thereby enhancing reactivation and cortical integration of declarative memories. Given the anatomical proximity of respiratory centres to brainstem noradrenergic nuclei, this work bridges bodily physiology with cognitive neuroscience and raises intriguing questions about potential interactions between breathing and LC-NA dynamics.

Finally, Yang et al. (2025) demonstrated in healthy humans that signatures of sleep-dependent memory processing are temporally coupled with peripheral glucose regulation. Using simultaneous polysomnography and continuous glucose monitoring, they found that sleep spindles were followed within minutes by a decrease in glucose, whereas slow oscillations were followed by an increase. This fine-tuned link suggests that the neurophysiological mechanisms of hippocampus-dependent memory consolidation are integrated with short-term metabolic regulation, possibly *via* noradrenergic pathways that modulate both neuronal activity and peripheral metabolism. The study provides direct human evidence for a close sleep-memory-metabolism triad, with implications for conditions like diabetes and sleep disorders and underscores the systemic reach of NREM sleep’s organising principles.

### N2–N3 division of labor in memory consolidation

N2 (light sleep phase) and N3 (slow-wave sleep) are not simply homogeneous in memory consolidation, but rather function partially separably and mutually complementary through their characteristic EEG rhythms: N2 phase, dominated by sleep spindles, primarily supports the fine stabilisation of specific memory traces (especially motor and perceptual learning); N3 phase, characterised by high-amplitude slow oscillations and abundant slow-wave activity, plays a key role in the reorganisation and cross-regional redistribution of newly acquired hippocampus-dependent memories to the neocortex. Beyond a purely stage-based perspective, contemporary models now highlight that specific oscillatory events within these stages-sleep spindles, slow oscillations and hippocampal sharp-wave ripples-and, critically, their cross-frequency coupling, are the proximal neurophysiological substrates of offline memory processing rather than sleep stages perse (Helfrich et al. 2019; Klinzing et al. 2019).

#### N2 spindle rhythmicity and temporal clustering

N2 is characterised by prominent sleep spindles in the sigma band. As mentioned earlier; The variations in spindle wave density, amplitude and terrain, as well as the accuracy of the coupling between spindle waves and cortical slow oscillations, can predict the consolidation of memory. N2 rich periods, even in the relative absence of high levels of slow wave activity, can be sufficient to yield measurable memory benefits in both motor sequence learning and perceptual learning paradigms.

Crucially, interventional studies have begun to establish a causal link between N2 spindle activity and memory consolidation. Closed-loop auditory stimulation timed to slow oscillation up-states enhances declarative memory by boosting spindle activity (Zhang et al. 2025). In the motor domain, sigma amplitude during stimulation—rather than slow oscillation parameters—predicts overnight procedural memory gains, suggesting distinct mechanisms for declarative and procedural memory. While tACS with spindle-like waveforms alone may not suffice, the density of naturally occurring spindles robustly correlates with motor memory consolidation, underscoring the importance of precisely-timed, physiologically-grounded interventions (Moyne et al. 2025). The critical role of spindle timing is further evidenced by real-time spindle tracking studies. Antony et al. (2018) showed that memory reactivation *via* auditory cues is effective only when cues fall outside the spindle refractory period (∼3–6 s post-spindle), demonstrating that spindle rhythmicity gates windows of opportunity for memory processing during N2 sleep. Motor learning increases N2 spindle density locally in task-relevant cortical regions, predicting overnight performance gains (Mylonas et al. 2017).

Beyond spindle density, the temporal organisation of spindles into ‘trains’ during N2 sleep has emerged as a key mechanism. Boutin and Doyon (2020) first proposed a spindle framework wherein the temporal clustering of spindles provides optimal conditions for interference-free memory reactivation. Recent empirical work has refined this view: Conessa et al. (2026) demonstrated that spindle trains—periodic clusters occurring at an infraslow rhythm (∼0.02 Hz)—promote skill-specific strengthening, whereas isolated spindles may facilitate skill generalisation by creating memory-instability conditions. This framework underscores that the clustering pattern, not merely spindle presence, shapes memory outcomes. Closed-loop auditory stimulation targeting spindles elucidate the neurophysiological consequences of such manipulations. Jourde et al. (2025) showed that stimulating spindles can either truncate an ongoing spindle (if stimulated early) or evoke subsequent sigma activity (if stimulated late), validating closed-loop approaches as tools to causally probe spindle function.

Collectively, these findings argue that N2 sleep, *via* its spindle activity, plays a direct and functionally specific role in stabilising and refining motor and perceptual memories.

#### N3 slow oscillations and the triple coupling framework

In contrast, N3 is primarily defined by high-amplitude slow oscillations (SOs) and elevated slow-wave activity. While the detailed architecture of SO-spindle-ripple triple couplinghas been thoroughly discussed in the previous text, here we focus on the distinct role of N3 in orchestrating this hierarchical interaction. The depolarising up-states of SOs create temporal windows of enhanced cortical excitability that synchronise thalamocortical spindles and, in turn, hippocampal ripples, enabling precisely timed information transfer from hippocampus to neocortex (Said and Miyamoto 2025). Computational modelling work has further elucidated that slow oscillations not only group these faster rhythms but also actively promote the reorganisation of memory traces by facilitating synaptic competition, which may serve to weaken less salient representations while strengthening those that are behaviourally relevant (Wei et al. 2018). Unlike the more localised spindle activity characteristic of N2, slow oscillations during N3 exhibit travelling wave properties that coordinate activity across distributed cortical networks, providing the global framework necessary for large-scale memory reorganization (Cox et al. 2018). Thus, N3 serves as the physiological scaffold within which the fine-grained, spindle-mediated refinement initiated during N2 can be integrated into broader neocortical networks.

#### N2 and N3: from strict stages to a multi-step consolidation process

The preceding sections have detailed the distinct contributions of N2 and N3 to memory consolidation. However, a growing body of evidence indicates that this division is not absolute, but rather reflects a dynamic and integrated multi-step process.

First, both stages can express the critical oscillatory motifs—spindles, slow oscillations and ripples—and it is their precise temporal coordination, rather than the macro architectural label of N2 versus N3, that best predicts memory outcomes (Chang et al. 2025). The hierarchical nesting of these oscillations provides a mechanistic basis for information transfer during sleep. Using direct intracranial recordings from the human hippocampus, Staresina et al. (2015) demonstrated that hippocampal ripples are nested in the troughs of sleep spindles, which in turn are modulated by the up-state of slow oscillations—establishing the triple coupling framework that enables precisely timed hippocampal-neocortical communication during NREM sleep. Studies directly comparing N2 and N3 sleep have further elucidated this point. Cox et al. (2018) demonstrated that while slow oscillations are spatially restricted, their phase modulates local spindle activity at virtually every cortical site, with coupling dynamics varying between spindle classes and sleep stages, and exhibiting stable individual fingerprints. Dehnavi et al. (2019) provided particularly compelling evidence for functional differentiation, showing that spindle power in N2 and SWS responds in opposite directions to motivated forgetting experiences: N2 spindle power negatively correlated with recall of to-be-forgotten items (supporting active forgetting), whereas SWS spindle power positively correlated with recall (supporting memory preservation). This opposite pattern underscores that spindles in N2 and N3 serve distinct, and sometimes opposing, functional roles. Moreover, Dehnavi et al. (2023) revealed that stronger slow-spindle to SO down-state coupling in N2 characterises individuals with higher general memory ability, whereas fast spindle properties predict overnight memory consolidation—further dissociating the contributions of these two stages.

Second, N2 and N3 often occur in close temporal proximity within NREM cycles, allowing memory traces to be iteratively processed across successive bouts of spindle-rich and slow oscillation-rich activity. As articulated in the active systems consolidation framework (Klinzing et al. 2019), this sequential architecture enables hippocampal replay during slow-wave sleep to gradually transform and integrate representations in neocortical networks, with the interplay of slow oscillations, spindles and ripples regulating information flow across distant brain networks. Computational modelling work has further shown that this sequence—wherein N2-associated spindle activity (allowing parallel, independent replay) precedes N3-associated slow oscillations (promoting synaptic competition)—is optimal for consolidating multiple competing memories without interference (Wei et al. 2018). The precise temporal organisation of spindles into ‘trains’ during N2 sleep (Conessa et al. 2026) may provide a framework for repeated, interference-free reactivation that primes memories for subsequent large-scale reorganisation during N3.

Finally, inter-individual differences in the relative amount and microstructure of N2 and N3 appear to shape which types of memories are most strongly consolidated during sleep and may partially account for developmental and ageing related changes in sleep-dependent memory. Helfrich et al. (2018) demonstrated that age-related gray matter atrophy in the medial prefrontal cortex disrupts the precise coupling of slow oscillations and fast spindles, leading to impaired overnight memory consolidation in older adults. This temporal ‘uncoupling’—wherein spindles arrive too early in the SO cycle—directly predicts the degree of forgetting. Muehlroth et al. (2019) further showed that older adults exhibit a shift from fast spindle-SO coupling towards increased slow spindle activity at the end of the SO up-state, and that this age-typical pattern already predicts worse memory consolidation in younger adults. Crucially, preserved structural integrity in medial prefrontal cortex, thalamus and medial temporal lobe regions promotes ‘youth-like’ coupling patterns in old age, reinforcing the link between brain maintenance and successful sleep-dependent memory consolidation.

Thus, rather than functioning as strictly segregated stages, N2 and N3 jointly implement a multi-step consolidation process in which N3-associated slow oscillations orchestrate hippocampo-neocortical information transfer, while N2-associated spindles refine and stabilise the resulting neocortical traces. This process is embedded in a global synaptic downscaling during sleep, with the precise coupling of oscillations—across both stages—serving as the key physiological substrate for enduring memory.

### Related REM-specific oscillatory phenomena

REM sleep is typically subdivided into two distinct microstates. Tonic REM represents the majority of REM sleep and is characterised by high cortical activity with EEG patterns resembling wakefulness (desynchronised low-amplitude fast rhythms), generalised muscle atonia and minimal eye movements (Blumberg et al. 2020). Interspersed within tonic REM are brief phasic REM episodes, marked by rapid eye movements and large-amplitude phasic field potentials known as ponto-geniculo-occipital (PGO) waves, which originate in the pons and propagate to the lateral geniculate nucleus (LGN) and occipital cortex (Ramirez-Villegas et al. 2021). Phasic REM is also associated with transient facial, limb, or digit twitches, and irregularities in respiration and heart rate.

From the mid-twentieth century onward, animal studies using diverse behavioural paradigms have shown that REM sleep facilitates memory consolidation. Selective REM deprivation in animals disproportionately impairs complex forms of learning-such as two-way shuttle-box avoidance or navigation in complex mazes-while having smaller effects on simpler tasks, such as one-way avoidance or simple mazes (Zager et al. 2007). Developmentally restricted REM deprivation in kittens produces long-lasting deficits in emotion-related memory circuits-including the LGN and amygdala-leading to altered adult social and affective behaviors (Blumberg et al. 2022). In humans, selective REM deprivation has little to no impact on declarative memory consolidation but significantly impairs procedural memory, particularly after complex skill acquisition. These findings led (Smith 2001) to propose that in humans, REM sleep preferentially subserves procedural/skill-based memory rather than declarative memory, especially when tasks are simple.

REM sleep appears to operate on this NREM‑stabilised substrate in a qualitatively different way. In REM, a highly cholinergic and aminergically quiescent milieu, combined with activated limbic and associative cortices and hippocampal theta–gamma coupling, favours the reorganisation rather than the faithful replay of prior experiences. REM has been preferentially linked to the consolidation of emotional memories, the extraction of gist and rules, the integration of new information into existing schemas, and the automatisation of certain motor skills. More recent work emphasises the role of REM sleep in emotional memory processing. REM alterations are common in psychiatric conditions characterised by stress or fear dysregulation-such as depression, anxiety disorders and PTSD-and often co-occur with sleep fragmentation, heightened reactivity to fear stimuli and recurrent nightmares (Baglioni et al. 2016). REM architecture disruptions can predict onset or progression of these disorders (Zhang et al. 2019). In addition, REM sleep has been implicated in social memory consolidation *via* neuropeptidergic mechanisms: during REM, oxytocin release in the hypothalamic paraventricular nucleus-prelimbic cortex pathway enhances VIP/PV interneuron activity, rebalancing microcircuit inhibition to drive social memory stabilisation (Liu et al. 2025).

#### Theta oscillations

EEG activity during REM sleep closely resembles that of wakefulness-hence the historical term paradoxical sleep. During tonic REM, robust 5–12 Hz theta oscillations are observed across the cortex, hippocampus and subcortical structures, with the hippocampus showing the most pronounced activity (Booth and Poe 2006). Theta acts as a ‘neural metronome’, temporally organising hippocampo–cortical communication; transient surges in theta power and frequency are associated with elevated firing rates and enhanced neuronal coordination across hippocampal and cortical networks (Peyrache et al. 2015; de Almeida-Filho et al. 2021). Building on this framework, recent work has begun to place REM theta within a broader architecture of state-dependent oscillations and large-scale network interactions, rather than treating it as an isolated hippocampal rhythm (Zielinski et al. 2020).

In humans, Nishida et al. (2009). first demonstrated that REM theta activity serves as a physiological marker of emotional memory consolidation: shorter latencies to REM onset correlated with stronger retention of emotionally salient memories. In rodents, Popa et al. (2010). showed that theta synchronisation across the amygdala–prefrontal cortex–hippocampus network during REM is critical for consolidating fear memories, with coupling strength predicting recall performance. Extending these correlational findings, causal manipulation studies have reinforced the necessity of intact REM theta. Boyce et al. (2016). used optogenetics to selectively suppress hippocampal theta oscillations during a defined 4-h post-learning REM window, which abolished consolidation of spatial and contextual fear memories without affecting cue-based fear or motivation-an effect absent when interventions targeted NREM or wake states.

At the same time, not all manipulations of REM-related rhythms yield uniform effects, underscoring the importance of frequency- and phase-specific mechanisms. Harrington et al. (2021). demonstrated that closed-loop auditory stimulation can transiently phase-lock or modulate theta during REM without altering emotional memory, suggesting that coarse perturbations of oscillatory phase are insufficient, and that more precise interference with endogenous hippocampal–amygdala–prefrontal coupling may be required. Complementing these findings, Torres-Herraez et al. (2022). identified cerebellar delta waves and pontine sleep potentials (PSPs) during REM that modulate hippocampal theta, providing a physiological substrate for cerebello–hippocampal dialogue in sleep-dependent consolidation. These results collectively argue that REM theta should be understood as embedded in a hierarchy of brainstem, cerebellar and forebrain events rather than as a unitary phenomenon.

From a computational perspective, several modelling studies have attempted to formalise how REM theta shapes memory traces. Timothy et al. (2022). incorporated REM-like theta dynamics into artificial neural networks, showing that the alternation of NREM-like ‘stabilization’ and REM-like ‘integration’ phases improved generalisation while preserving key episodic details. In related work, Rho et al. (2023). modelled the impact of 4 Hz versus 10 Hz theta input to prefrontal–amygdala circuits during REM. They found that 4 Hz theta strengthened prefrontal–amygdala connectivity and suppressed fear-related outputs, whereas this GABAergic suppression failed under simulated PTSD-like conditions with elevated norepinephrine. Intriguingly, 10 Hz theta restored suppression in the model, suggesting that the frequency of REM theta may differentially regulate emotional salience and might be harnessed therapeutically. Finally, Schott et al. (2023). identified dorsomedial medullary CRH neurons as dual regulators of REM and PGO-like waves; their excitatory projections promote REM, increase PGO density and enhance hippocampal theta bursts, thereby providing a mechanistic link between neuromodulatory control of REM architecture and the oscillatory signatures thought to support consolidation.

These experimental and computational findings support a more integrated view in which REM theta operates within a multi-oscillatory, multi-regional network. Human and animal studies converge in showing that REM theta is tightly coupled to emotional memory strength, while causal perturbations demonstrate that selectively disrupting hippocampal theta during REM impairs consolidation in a content- and state-dependent manner. Yet the mixed effects of external stimulation, and the modulatory roles of cerebellar and brainstem generators, caution against overly simplistic accounts. Instead, REM-dependent consolidation appears to rely on the precise timing, frequency and regional coordination of theta with other sleep events-constraints that recent network models begin to capture. Within this broader context, REM can be distinguished from, but not separated from, NREM: theta-dominated REM episodes do not merely ‘replay’ memories but reweight their emotional significance and network embedding against a background previously shaped by NREM-linked spindles and slow oscillations.

#### Ponto-Geniculo-occipital waves

Ponto-Geniculo-Occipital (PGO) Waves, originally recorded in cats in the 1950s, PGO waves are sharp, biphasic field potentials (200-300 μV, 60-120 ms) that originate in the pons, propagate to the LGN and terminate in the occipital cortex (Arnulf et al. 2018). They typically occur in bursts during phasic REM but can also emerge sporadically during NREM-REM transitions (Gao et al. 2023). While initially described in felines, PGO-like events have since been reported in rodents, primates and possibly humans-fMRI and polysomnography data reveal occipital cortex activation during REM even in the absence of retinal input (Miyauchi et al. 2009).

Numerous studies have demonstrated that PGO waves occurring during REMs are closely associated with the process of memory consolidation (Hutchison and Rathore 2015).

In rodents, post-learning increases in PGO density and frequency have been consistently observed (Datta 2000; Datta et al. 2004). Pharmacological enhancement of PGO activity can rescue avoidance memory deficits caused by REM deprivation (Mavanji et al. 2004), while suppression of PGO generation impairs retrieval. PGO density can also predict the success of fear extinction training (Datta and O’Malley 2013). Transition-phase PGO waves, which co-occur with hippocampal SWRs at frequencies lower than those of phasic REM, may act as triggers for REM onset. Phase alignment between PGO peaks and hippocampal theta has been proposed to induce long-lasting synaptic potentiation, enabling reinforcement of new memories without overwriting existing traces (Karashima et al. 2001).

Recent work implicates dorsomedial medullary CRH-expressing neurons as an upstream hub coordinating both PGO generation and REM maintenance (Schott et al. 2023). Activation of these neurons increases PGO occurrence, enhances theta and promotes memory consolidation, while their suppression has the opposite effects. Although PGO waves have been linked to learning, dreaming, visuomotor integration and synaptic plasticity in implicated cortical and subcortical regions, their precise computational role in memory processing remains to be fully elucidated.

### Synaptic remodeling during REM sleep

The neural regulation of REM sleep originates predominantly from brainstem nuclei, which release acetylcholine to activate the thalamus and cortex, producing a desynchronised EEG pattern. Concurrently, noradrenergic neurons in the locus coeruleus and serotonergic neurons in the raphe nuclei exhibit markedly reduced activity during REM, creating a distinct neurochemical milieu. This unique pattern of elevated cholinergic tone and suppressed monoaminergic signalling provides optimal conditions for synaptic plasticity (Aime et al. 2022).

During both neural development and cognitive learning, REM sleep contributes to the pruning of dendritic spines at newly formed synapses (Li et al. 2017). Wagner et al. reported that REM sleep episodes predominantly occurring in the latter half of the night confer a mnemonic advantage, preferentially extending the retention of emotional memories over neutral ones (Wagner et al. 2006). Walker and colleagues formulated the influential ‘sleep to forget and sleep to remember’ hypothesis, which posits that emotional memories comprise two separable components: informational content and the associated affective charge. During sleep, particularly REM, these components are progressively decoupled-allowing consolidation of factual content while attenuating its emotional salience (Walker 2009).

Mechanistically, Wei Li et al. demonstrated in mice that selective REM deprivation abolishes NMDA receptor-driven dendritic calcium waves that normally promote spine remodelling-both pruning and strengthening-during REM. This leads to a marked increase in the elimination of nascent spines and the selective long-term stabilisation of a small subset of functionally crucial spines, a process essential for circuit refinement and behavioural optimization (Li et al. 2017). Similarly, Zhou et al., using targeted sleep interventions combined with high-resolution *in vivo* imaging, established REM as a primary driver of experience-dependent dendritic spine elimination, mediated by amplified dendritic calcium spikes. In paradigms such as visual deprivation and fear conditioning, REM facilitated synaptic refinement and downregulation of neuronal excitability, thereby directly supporting memory consolidation (Zhou et al. 2020).

In the hippocampal dentate gyrus, Kumar et al. found that adult-born neurons (ABNs) activated during learning undergo selective reactivation during REM. Although overall ABN population activity was reduced, a sparse subset preserved precise reactivation patterns. Optogenetic silencing of ABNs during REM impaired memory consolidation, lengthened spine necks, downregulated synaptic plasticity-related gene expression and weakened fear memory retention. This effect was temporally and spatially specific: only recently generated ABNs engaged in sparse REM reactivation contributed to consolidation by coordinating spine remodelling and transcriptional programs (Kumar et al. 2020). Providing causal validation, Srinivasan et al. revealed that reactivation of as few as 2–3 recently recruited ABNs during the ascending phase of hippocampal theta oscillations in REM was both necessary and sufficient for consolidating fear memories; identical silencing during wakefulness had no effect. This specificity extended to cell type and learning episode-mature neurons or unrelated ABNs played no role. These findings highlight a temporally gated, theta-driven ‘micro-ensemble reactivation’ strategy through which the brain efficiently stabilises memory traces, underscoring the interplay between sparse coding and neuronal timing precision (Srinivasan et al. 2025).

### Complementarity of NREM and REM in memory consolidation

Most empirical and theoretical work now converges on the idea that memory processing during sleep is not confined to a single stage, but instead emerges from the dynamic interaction between NREM and REM across recurrent sleep cycles. In one influential framework, NREM-particularly N3-supports the reactivation and redistribution of newly encoded hippocampus dependent memories. During slow wave–rich N3, hippocampal sharp wave ripples are temporally nested within cortical slow oscillations and spindles, driving the gradual strengthening of neocortical representations. Subsequent REM episodes are then proposed to promote synaptic consolidation, integration and abstraction, possibly *via* heightened cholinergic tone, desynchronised cortical activity and the re-activation of distributed associative networks (Hobson and Pace-Schott 2002; Walker and Van Der Helm 2009; Boyce et al. 2016). Evidence for such complementary processing includes findings that:

1. The amount and sequence of NREM and REM across the night often predict memory outcomes better than any single stage alone (Walker and Stickgold 2004; Lee et al. 2011; Perez-Ruiz and Herrero-Beites 2014).
2. Targeted memory reactivation during NREM can bias which memories are later re activated during REM (Miyo et al. 2016; Djurdjevic et al. 2018).
3. Emotional memories, in particular, appear to benefit from an initial NREM dependent strengthening of factual content, followed by REM related recalibration of affective responses (Gago et al. 2014).
4. NREM and REM sleep play distinct but complementary roles in synaptic reorganization (Schott et al. 2023). NREM primarily promotes synaptic potentiation, strengthening connections that support memory encoding and storage. REM instead facilitates synaptic pruning, weakening or eliminating less relevant connections to refine memory networks (Arnulf et al. 2018). This balance between synaptic strengthening and weakening underlies memory consolidation, enhancing learning capacity, cognitive flexibility and the long-term stability of stored information.

These observations suggest that considering NREM and REM in isolation may underestimate the importance of their interaction. N2/N3 driven systems consolidation and REM related synaptic and emotional processing may form a recurrent, multi-cycle algorithm (Feld and Born 2020), in which each cycle iteratively refines memory traces and their integration into existing knowledge structures (Jones 2020) (Table 2).

**Table 2.** Representative studies on how distinct sleep stages and their characteristic EEG oscillations contribute to memory consolidation.

Sleep stage	EEG indicators	Author (years)	Models	Memory type/main research methods	Main findings
NREM	SO	Ngo et al. (2013); Ngo and Staresina (2022) Weinhold et al. (2022)	Human	Declarative (verbal)	The UP state of SOs is the optimal window for triggering memory reproduction and consolidation.
		Nicolas et al. (2025)		Procedural	Up state enhance SOs and optimise striato-motor network activity.
		Marshall et al. (2006)		Declarative and Procedural	Enhancing SOs potentiates memory consolidation.
		Massimini et al. (2007)		TMS	TMS triggers SOs revealing thalamocortical bistability.
		Mittermaier et al. (2024)		Multineuron patch-clamp	Up / Down states enhance synaptic consolidation by modulating axonal action potentials.
	SWR	Staresina et al. (2015)	Human (Epilepsy)	Declarative (episodic)	Reveals hierarchical nesting of slow oscillations, spindles and ripples in human hippocampus during NREM sleep for memory consolidation.
		Schreiner et al. (2024)			Spindle-locked medial temporal lobe ripples, coupled with slow oscillations, mediate episodic memory reactivation during human NREM sleep.
		Zhang et al. (2018)			The replay strength of SWRs can predict next-day memory retention.
		Logothetis et al. (2012)	Monkey	Declarative (episodic)	Hippocampal ripples in monkeys drive cortical activation and subcortical suppression, suggesting off-line consolidation of episodic declarative memory.
		Girardeau et al. (2009)	Rat	Declarative (spatial)	Closed-loop disruption of SWRs impaired produced severe deficits in spatial memory.
		Fernández-Ruiz et al. (2019)			Precise regulation of SWRs can control memory performance.
		Giri et al. (2024)			The discharge of excitatory RSC neurons precede SWRs, while inhibitory neurons follow.
		Rothschild et al. (2017)		Declarative (associative)	The first demonstration of a cortico-hippocampal-cortical information flow loop during SWRs.
		Oliva et al. (2016)		*In vivo* electrophysiology	CA2 pyramidal neurons initiate hippocampal sharp-wave ripples before CA3 and CA1, with deep ramping and superficial phasic sublayer cells.
		Karimi Abadchi et al. (2020)	Mouse	Declarative (spatial)	The effects of SWRs playback in different states on neocortex activity vary.
		Grosmark et al. (2021)			Hippocampal neuronal reactivation during post-exploration sleep directly predicts the consolidation of unbiased long-term spatial cognitive maps in adult male rats.
	Spindle	Cairney et al. (2018)	Human	Declarative (episodic)	Memories newly formed prior to a nap selectively triggered rapid increases in spindle activity.
		Petzka et al. (2022)			The spatial distribution of spindles corresponded closely to cortical regions engaged during encoding.
		Schreiner et al. (2022, 2024)			Discover and emphasise the hierarchical coupling of SO-spindle-ripple as the core mechanism.
		Cross et al. (2025)		Declarative (verbal)	SO–spindle coupling is a stable individual trait and highly sensitive to encoding depth.
		Sjøgård et al. (2025)		Procedural	Cortical areas active during motor-sequence learning showed more regular neural activity in subsequent sleep.
		Baena et al. (2022)		EEG-fMRI analysis	SO–spindle coupling preferentially drove hippocampal reactivation.
		Duan et al. (2025)	Human (Epilepsy)	Declarative (spatial)	The study reveals that memory consolidation during sleep involves dynamic hippocampal-cortical coupling followed by decoupling.
		Dickey et al. (2024)		EEG analysis	Spindles and SO UP states coordinate corticohippocampal high-frequency co-ripple events.
		Mak-McCully et al. (2017)			SOs DOWN states trigger spindle bursts, transmitted to the cortex during transition.
		Schreiner et al. (2021)		iEEG-EEG analysis	ATN ensure spindle events precisely nested within cortical SO UP states.
		Li et al. (2024)			SWRs act as binary triggers for transient plasticity, jointly promoting consolidation with spindles.
		Wang et al. (2023)	Rat	Emotional	Spindle strengthen memory representations in target regions, enhancing associative memory by reinforcing integrative hubs.
		Kim et al. (2019)		Procedural	Use optogenetic inhibition to show SO-spindle disruption impairs consolidation, while delta disruption promotes forgetting, revealing competing roles.
		Toader et al. (2023)	Mouse	Declarative (episodic)	AM is a gating hub for long-term memory consolidation, coordinating thalamo–cortical loops to mediate hippocampo–cortical transfer and stabilisation.
		Alizadeh et al. (2022)		Declarative (spatial)	Long, low-frequency spindles markedly increased hippocampo–thalamic synchrony.
		Tononi and Cirelli (2003)	Human and Rat and Mouse and Cat	Procedural	Sleep downscales synapses *via* slow waves to enhance learning and performance.
		Barthó et al. (2014)	Rat and Mouse	*In vivo* electrophysiology	The duration and amplitude of spindle events are determined by thalamo–cortical interactions.
		Seibt et al. (2017)		Calcium imaging techniques	SOs high-frequency components at UP-state peaks modulate dendritic Ca²⁺ influx to optimise synaptic plasticity.
REM	θ Oscillations	Nishida et al. (2009)	Human	Emotional(fear)	First demonstrated that REM theta activity is a physiological marker of emotional memory consolidation.
		Harrington et al. (2021)			Closed-loop auditory stimulation could transiently phase-lock or modulate theta without altering emotional memory, underscoring frequency-specific effects.
		Popa et al. (2010)	Rat		theta synchronisation across the amygdala–prefrontal cortex–hippocampus network during REM is critical for consolidating fear memories.
		de Almeida-Filho et al. (2021)			Enhanced HPC-RSC interaction during phasic REM sleep promotes fear memory consolidation.
		Zielinski et al. (2020)		Declarative (spatial)	Hippocampal replay and theta sequences interact with prefrontal cortex to support memory-guided behaviour.
		Schott et al. (2023)		*In vivo* electrophysiology	A common medullary hub of CRH neurons controls REM sleep and promotes P-waves, linking brainstem activity to phasic REM events.
		Boyce et al. (2016)	Mouse	Declarative (episodic and Emotional)	Suppress hippocampal theta oscillations during REMs, which abolished consolidation of spatial and contextual fear memories
		Peyrache et al. (2015)		Declarative (spatial)	The head direction system is maintained by an internal attractor mechanism during sleep, onto which sensory inputs are grafted for precision and updating.
		Torres-Herraez et al. (2022)		*In vivo* electrophysiology	Cerebellar delta waves and pontine sleep potentials (PSPs) during REM that modulate hippocampal theta.
		Schott et al. (2023)			CRH neurons excitatory projections promote REM, increase PGO density and enhance theta bursts.
		Timothy et al. (2022)		SRC simulation	Incorporated REM theta activity into replay-based learning algorithms, mitigating catastrophic forgetting in artificial neural networks.
		Rho et al. (2023)		Biophysical model	4 Hz theta input during REM strengthened prefrontal–amygdala connectivity and suppressed fear memories.
	PGO waves	Ramirez-Villegas et al. (2021)	Macaca	Declarative	Transition-phase PGO waves, which co-occur with hippocampal SWRs at frequencies lower than those of phasic REM.
		Hutchison and Rathore (2015)	Rat	Declarative (spatial)	Hippocampal replay and theta sequences mediate prefrontal interactions to support spatial memory encoding, retrieval and planning.
		Datta (2000); Datta et al., (2004); Datta and O’Malley (2013)		Procedural and Emotional	From phenomenon discovery to mechanism verification and then to functional expansion, pgo waves are a necessary condition for the successful consolidation of emotional memory.
		Mavanji et al. (2004)		Emotional	Pharmacological enhancement of PGO activity can rescue avoidance memory deficits caused by REM deprivation.
		Carrera-Cañas et al. (2019)	Cat	*In vivo* electrophysiology	PGO activity and theta may be regulated by a common mechanism.
		Karashima et al. (2001)			Phase alignment between PGO peaks and hippocampal theta can induce long-lasting synaptic potentiation.

The table is organised by sleep stage (NREM/REM) and core EEG markers (slow oscillations, sleep spindles, hippocampal sharp-wave ripples, REM theta and PGO waves). For each study, it lists the sample, memory domain or paradigm, and main findings. Overall, NREM SO–spindle–ripple coupling supports systems consolidation and synaptic renormalisation, whereas REM theta and PGO activity are more involved in emotional and procedural memory, contextual integration and synaptic remodelling, underscoring complementary NREM–REM contributions.

## Conclusions

Contemporary systems consolidation models conceptualise memory not as a static transfer from hippocampus to neocortex, but as the gradual reorganisation of a distributed engram network. Recent episodic memories are rapidly encoded in sparse, plastic engram cells within the hippocampal–entorhinal circuit, while weaker, parallel engram traces are ‘pre-tagged’ in prefrontal, amygdalar and mesolimbic structures. During subsequent offline periods, particularly sleep, coordinated hippocampo–cortical reactivation strengthens neocortical and medial prefrontal engrams and their cortico–cortical connections, supporting the emergence of more stable, abstract and schema-like representations. In parallel, interactions with the amygdala and dopaminergic midbrain–striatal systems assign emotional valence and motivational salience, thereby biasing which traces are preferentially stabilised (Berry et al. 2015). Over time, the spontaneous retrieval of remote memories becomes increasingly supported by cortical (especially mPFC-centered) networks, although the hippocampus can be re-engaged during recall to mediate systems reconsolidation: new hippocampal engrams are recruited and integrated with existing cortical traces, enabling the updating of remote memories without erasing their prior structure. Thus, systems consolidation and reconsolidation reflect ongoing, time-dependent reactivation, reweighting and partial reconfiguration of a multi-node engram network spanning hippocampal, entorhinal, prefrontal, amygdalar and reward-related circuits (Hughes 2020).

Distinct stages of sleep employ specialised neural activity patterns to synergistically promote memory consolidation. During non-rapid eye movement sleep (NREM), cortical slow oscillations (SOs) create UP/DOWN state alternations that serve as temporal windows for cross-regional information transfer. SOs, when nested with thalamic spindles, coordinate hippocampal sharp-wave ripples (SWRs), driving the transfer of labile hippocampal engrams to cortical networks. The hierarchical phase coupling of ‘SOs-spindles-SWRs’ facilitates the spatiotemporal reorganisation of mnemonic representations. At the synaptic level, NREM supports a dynamic balance between global synaptic downscaling-optimising network efficiency-and local plasticity enhancement, which selectively strengthens memory-relevant synapses.

In REM sleep, theta oscillations synchronise limbic-prefrontal-hippocampal circuits, while phase-locked PGO waves further integrate emotional memory traces and procedural skills. The characteristic high-cholinergic, low-monoaminergic neurochemical environment fosters dendritic spine remodelling and sparse neuronal reactivation, enabling the dissociation of memory content from its affective load and the targeted restructuring of synaptic connections.

Despite significant advances, several key questions remain. First, the cross-scale regulatory mechanisms underlying SO-spindle-SWR coupling are incompletely understood due to a lack of technologies capable of dynamically tracking hippocampal-cortical transfer pathways *in vivo*. Second, the precise manner in which REM theta-PGO coupling modulates synaptic plasticity awaits verification using simultaneous multi-region recordings. Third, while REM is known to bidirectionally regulate the pruning and stabilisation of nascent spines, the cellular and molecular underpinnings of this dual control remain elusive. Fourth, current research has predominantly focused on neuronal reactivation; the potential ­involvement and functional contributions of glial cells to sleep-dependent consolidation remain largely unexplored-including whether neuronal replay modulates glial behaviour. Lastly, translation of sleep-memory findings into clinical interventions lags behind mechanistic discoveries. Most rhythm-based memory enhancement strategies remain at experimental stages, with little adaptation to pathological contexts.

In summary, research on sleep EEG oscillations has moved the field beyond a purely stage-based description of sleep towards an event-based, mechanistic framework. Specific oscillatory motifs and their cross-frequency coupling—most prominently slow oscillations, spindles, hippocampal ripples, REM theta and PGO waves—are now viewed as proximal operations that gate hippocampo-cortical communication and synaptic plasticity during memory consolidation. Nevertheless, the causal mapping between particular oscillatory patterns and distinct memory functions remains incomplete, and glial, neuromodulatory and metabolic contributions to sleep-dependent plasticity are still under-characterised. Future multiscale and computationally informed studies will be essential to address these gaps and to translate sleep EEG markers into mechanistically grounded, clinically relevant tools.

Future research should also integrate high-density EEG with AI-driven analytics to derive quantifiable biomarker profiles for portable monitoring and early detection of sleep-related cognitive risk. Such tools could accelerate the development of clinically viable assessment strategies and targeted interventions tailored to an individual’s sleep architecture.
